# Editorial: Obesity and nutrition in the most remote parts of Africa

**DOI:** 10.3389/fpubh.2023.1197367

**Published:** 2023-05-25

**Authors:** Abdullahi Tunde Aborode, Agwuna Favour Obianuju, Helen Onyeaka, Ayoola S. Fasawe, Grace Adeola Adegoye, Christain Oko, Henry Chukwuemeka Uro-Chukwu

**Affiliations:** ^1^Department of Chemistry, Mississippi State University, Starkville, MS, United States; ^2^Healthy Africans Platform, Research and Development, Ibadan, Nigeria; ^3^Faculty of Pharmaceutical Sciences, University of Nigeria, Enugu, Nigeria; ^4^School of Chemical Engineering, University of Birmingham, Birmingham, United Kingdom; ^5^School of Biological Sciences, Illinois State University, Normal, IL, United States; ^6^Department of Nutrition and Health Science, Ball State University, Muncie, IN, United States; ^7^Division of Health Research, University of Lancaster, Lancaster, United Kingdom; ^8^Department of Community Medicine, Ebonyi State University, Abakaliki, Nigeria

**Keywords:** intersection, public health, Africa, obesity, remote

Obesity has long been recognized as a significant contributor to the development of many chronic diseases and their negative health effects; it raises the risk of many illnesses and conditions associated with an increased mortality rate. Numerous health conditions are linked to obesity, such as Type 2 diabetes mellitus (T2DM), cardiovascular diseases (CVD), metabolic syndrome (MetS), chronic kidney disease (CKD), hyperlipidaemia, hypertension, non-alcoholic fatty liver disease (NAFLD), certain types of cancer, obstructive sleep apnoea, osteoarthritis, and depression (1). The treatment of these ailments contributes to the burden on healthcare systems, and the cost of treating them can be substantial. For instance, research estimates that obese individuals spend around 30% more on medical expenses compared to people with a normal body mass index (BMI) (1).

According to recent data from the World Health Organization (WHO), globally, more than 1 billion people have obesity−650 million adults, 340 million adolescents, and 39 million children (2), hence, making the prevalence of obesity an issue not just in developed nations but also spreading there. A new World Health Organization (WHO) analysis predicts that by December 2023, one in five adults and one in 10 children and teenagers in 10 high-burden African countries will be obese if strong action is not taken to halt the trends (2).

The prevalence of fat and overweight is increasing throughout Africa (3). This situation is a time bomb. Dr. Matshidiso Moeti, WHO Regional Director for Africa, warned that millions of people, including children, risk living shorter lifetimes as a result of the burden of poor health (3). This sharp increase in obesity rates also suggests that chronic non-communicable illnesses and communicable diseases now coexist in Low-Income Countries (LICs), placing LICs under a double burden. Controlling obesity has become one of the top objectives for public health practitioners in developed countries because it is primarily caused by a long-term energy imbalance between calories consumed and calories expended.

Obesity may be caused by a number of different processes. According to conventional knowledge, the primary reason is that the body has a lot more excess energy stored than it has used up. The extra energy is retained in fat cells, leading to the development of the typical obesity pathology. The obesity-causing nutrient cues will change due to the pathologic expansion of fat cells. The most recent study revealed that, for both weight management and disease prevention, the quality and food sources of nutrients matter more than their quantities in the diet. Against the backdrop of the interplay between nature and nurture, genetics and epigenetics, environment and micro-environment, an increasing number of factors are being uncovered as potential causes or contributors to obesity (1, 3).

In addition to the recognized role of gut dysbiosis in obesity development, research is shedding light on how impaired glucose and lipid metabolism can lead to secondary health complications, how food cravings are heightened in the brains of obese individuals, and how gut hormones, adipose tissue, and gut microbiota regulate appetite and satiety in the hypothalamus (4).

A person's propensity for weight gain is also known to be significantly influenced by genetic variables. Recent epigenetic studies have offered a number of very helpful methods for comprehending the global rise in obesity (4). This further shown that the connections between genetics, epigenetics, and environment concerning obesity and the functions of epigenetic variables in regulating metabolism, obesity risk, and its complications (4).

In this context the importance of breastfeeding in infancy cannot be overstated. Many benefits accrue from being breastfed, and promotion of exclusive breastfeeding in African countries is an important and viable preventive measure to help counter the current obesity epidemic. As global rates of overweight continue to rise, the role of breastfeeding in prevention is critical. Children who are never breastfed or who breastfeed for a short period of time have a higher risk of childhood obesity than those who breastfeed for 6 months (5). A 2015 meta-analysis calculated that those who were breastfed had a 26 percent reduced chance of overweight, and found that even adults who were breastfed are at reduced risk of overweight and obesity (6).

Attention has been focused on community-based approaches and social marketing campaigns as the most suitable form of intervention by public health professionals in the absence of high-risk approaches that are safe, effective, and widely available (such as drugs and surgery). However, the substantial efficacy of such interventions is only weakly supported by the available research. Accordingly, there should be an urgent need for proper infrastructure and research design to facilitate public health and mitigate this obesity in remote areas of Africa.

With this in mind, it is germane to address lifestyle risk factors like low physical activity and poor dietary pattern and quality, which are typically attempted as the initial strategy for helping the obese lose weight. Thus, the World Health Organization (WHO) recommends reducing total fats and free sugars they consume while increasing their diet of fruits, veggies, legumes, whole grains, nuts, and polyunsaturated fats (7).

The World Health Organization (WHO) advocates a range of crucial actions to address the issue of obesity and overweight. These include government regulations such as enforcing limits on the sugar content of food, fiscal policies such as levying taxes on sugar-sweetened beverages, food marketing regulations requiring manufacturers to reveal nutritional information, promoting healthier food options for young children and infants, creating safe facilities for physical activity and transport, and enhancing public health services (8).

Interestingly, Kenya, Tanzania, and Uganda are receiving assistance from the WHO, the International Development Law Organization, the International Development Research Centre, and the Swiss Development Cooperation as part of a global initiative to develop and implement legislative standards and financial incentives to support a healthy diet and regular exercise. Additionally, the WHO is partnering with 10 other African nations with high obesity rates to accelerate efforts to reduce obesity, which will aid in the prompt intervention of obesity in remote African regions (3, 8).

Finally, the current drug therapies for obesity seem to have limited long-term benefits and be linked with negative side effects. Recently, two of the most commonly used agents were taken off the market due to significant negative side effects (9). The body implements compensatory responses to achieve a positive energy balance in response to decreased food intake during weight loss via dieting or medication. Obesity or bariatric surgery functions by avoiding these compensatory reactions, causing satiety to set in after a tiny amount of food is consumed, and maintaining a negative energy balance. Surgery has proven to be significantly more effective than drug treatment. Long-term research has demonstrated that surgery can reverse type 2 diabetes, enhance cardiovascular risk factors, and significantly lower mortality (6, 8). As obesity-related health complications escalate, the total healthcare costs for patients double every decade, posing a costly challenge especially on children during COVID-19 pandemic (7, 9).

However, there is a need to address lifestyle risk factors like low physical activity and poor dietary pattern and quality as the initial strategy for helping the obese lose weight. There is need to reduce the total fats and free sugars people consume while increasing their diet of fruits, veggies, legumes, whole grains, nuts, and polyunsaturated fats.

Implement government regulations such as mandatory limits on food sugar content, fiscal policies such as taxing sugar-sweetened beverages, and food marketing regulations such as the requirement that manufacturers disclose their product's nutritional information. Also, promoting healthier foods for older infants and young children can reduce the influence of obesity in most remote part of Africa. Creating facilities for safe, active transport and recreation, strengthen public health services, conduct further research to aid in facilitating public health roles and to mitigate obesity in remote areas of Africa, developing and implementing legislative standards and financial incentives to support a healthy diet and regular exercise. By implementing these recommendations, we can combat obesity and overweight in the most remote part of Africa and help prevent the negative health effects associated with these conditions.

## Author contributions

AA conceived the idea. All authors wrote and edited the manuscript, contributed to the article, and approved the submitted version.
